# Multifunctional Bionic Periosteum with Ion Sustained‐Release for Bone Regeneration

**DOI:** 10.1002/advs.202403976

**Published:** 2024-09-03

**Authors:** Junjie Mao, Zhenqian Sun, Shidong Wang, Jianqiang Bi, Lu Xue, Lu Wang, Hongliang Wang, Guangjun Jiao, Yunzhen Chen

**Affiliations:** ^1^ Liquid‐Solid Structural Evolution & Processing of Materials (Ministry of Education) School of Materials Science and Engineering Shandong University Jinan Shandong 250061 P. R. China; ^2^ Department of Orthopaedics Qilu Hospital of Shandong University Jinan Shandong 250012 P. R. China; ^3^ The First Clinical Medical School Shandong University Jinan Shandong 250012 P. R. China; ^4^ Musculoskeletal Tumor Center Peking University People's Hospital Beijing 100044 P. R. China; ^5^ Shandong Second Medical University Weifang Shandong 261000 P. R. China; ^6^ Shanxian Central Hospital Heze Shandong 274300 P. R. China

**Keywords:** angiogenesis, anti‐inflammatory, bioactive glass fibre membrane, bionic periosteum, delayed ions release, osteogenesis, porous structure

## Abstract

In this study, a novel bionic periosteum (BP)‐bioactive glass fiber membrane (BGFM) is designed. The introduction of magnesium ion (Mg^2+^) and zinc ion (Zn^2+^) change the phase separation during the electrospinning (ES) jet stretching process. The fiber's pore structure transitions from connected to closed pores, resulting in a decrease in the rapid release of metal ions while also improving degradation via reducing filling quality. Additionally, the introduction of magnesium (Mg) and zinc (Zn) lead to the formation of negative charged tetrahedral units (MgO_4_^2−^ and ZnO_4_^2−^) in the glass network. These units effectively trap positive charged metal ions, further inhibiting ion release. In vitro experiments reveal that the deigned bionic periosteum regulates the polarization of macrophages toward M2 type, thereby establishing a conducive immune environment for osteogenic differentiation. Bioinformatics analysis indicate that BP enhanced bone repair via the JAK‐STAT signaling pathway. The slow release of metal ions from the bionic periosteum can directly enhance osteogenic differentiation and vascularization, thereby accelerating bone regeneration. Finally, the bionic periosteum exhibits remarkable capabilities in angiogenesis and osteogenesis, demonstrating its potential for bone repair in a rat calvarial defect model.

## Graphical abstract

Co‐existence of Mg^2+^ and Zn^2+^ can affect the phase separation during electrospinning, leading the transformation of fibre pore from connected to closed. Mg and Zn form negative charged tetrahedral units (MgO_4_^2−^ and ZnO_4_^2−^) in the glass network, locking in metal ions. Hence, bioactive glass fibre membrane with delayed metal ions release is prepared to promote bone formation as bionic periosteum.

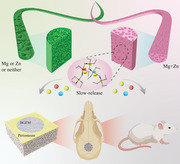

## 1 Introduction

The management of bone defects resulting from trauma and surgical procedures continues to pose significant clinical challenges. Such defects impose a substantial physical and financial burden on patients.^[^ ^1^ ^]^ As a vascularized fibrous membrane, periosteum is the connective tissue that covers the outer surface of all cortical bone and contributes more than 70% to the bone and cartilage regeneration potential.^[^ ^2^ ^]^ During the growth, development, and remodeling of bone, the periosteum provides growth factors, nutrients, and progenitor cells needed for proliferation or differentiation of cells.^[^ ^3^ ^]^ Therefore, protecting the integrity of periosteum during operation is of great significance to bone regeneration after operation. However, the bone defect caused by trauma is often accompanied by periosteal defect or contamination. Thus, the periosteal need to be resected but this may affect the bone repair process after operation.^[^ ^4^ ^]^ Therefore, an artificial periosteum material need to be urgently developed for clinical application. To mimic the biological function of natural periosteum, an ideal periosteal material should have immunomodulatory effects, promote osteogenesis and vascularization. However, most of the previous studies lacked these additional functions and thus hindered the bone repair process.^[^ ^5^ ^]^ The material should also have some degree of flexibility, act as a protective barrier and provide physical support to the damaged bone.

An ideal bionic periosteum (BP) serves a cell attachment scaffold that allows mineralized matrix deposition, designed to simulate the extracellular matrix (ECM) role during tissues formation.^[^ ^6^ ^]^ Among the multitudinous methods, ES can mimic the natural ECM making it suitable for the fabrication of porous continuous nanofiber scaffolds with high surface areas and more binding sites. This establishes a suitable environment and the necessary network that supports cell attachment, proliferation and differentiation.^[^ ^7^ ^]^ Currently, most studies on ES BP have focused on combining biocompatible polymer fiber membranes with inorganic nanoparticles,^[^ ^8^ ^]^ anti‐inflammatory drugs,^[^ ^9^ ^]^ or cytokines.^[^ ^10^ ^]^ However, the BP based on polymer fiber membrane are limited by high toxicity of crosslinking agents and rapid degradation, considering that the periosteum‐guided bone repair time can be as long as 6 months.^[^ ^10^, ^11^ ^]^ Notably, a bioceramic fiber membrane obtained by heat treatment can avoid the above shortcomings and provide long‐term mechanical support to resist the compression force exerted by the surrounding tissues during bone repair.^[^ ^12^ ^]^ In general, the preparation process of bioceramic fiber membranes is relatively complex and the obtained membranes lack the flexibility and strength required by BP.^[^ ^13^ ^]^

The poor mechanical characteristics of bioceramic fiber membranes stem from two primary factors. First, the formation of crystalline phases during calcination leads to low connectivity between grains and creates anisotropic stresses within the ceramic fibers. However, bioactive glass (BG) circumvents this issue by virtue of its amorphous silica composition, which offers high network connectivity.^[^ ^14^ ^]^ BG is a collection of [SiO_4_] tetrahedral units connected by the Oxygen atoms at the vertex. Therefore, Silicon (Si) is the forming atom of the glass network, which creates bridging oxygen (BO) bonds. Calcium (Ca) is the network modifier, disrupting networks by forming non‐bridging oxygen (NBO) bonds.^[^ ^15^ ^]^ Due to the asymmetry of glass network former and modifier, the degradation rate of two atoms is different. Si is gradually released from the glass matrix as silicic acid (Si(OH)_4_) owing to its covalent bonding, whereas Ca is rapidly released through ionic interactions with O.^[^ ^16^ ^]^ Challenges such as quick dissolution, sudden release of inorganic ions, and limited degradation limit the clinical utilization of BG.^[^ ^17^ ^]^ Thus, promoting the release of Si and decreasing the release of Ca can be an effective approach of improving BG. Previous studies have shown that incorporating specific metal cations into the BG matrix can significantly impact its physical properties, including network structure and thermal behavior.^[^ ^15^, ^18^ ^]^ For instance, both Magnesium (Mg) and Zinc (Zn) in BG structure can act as either a network modifier or a network former, which is in the form of intermediate oxide similar to the role of Si.^[^ ^19^ ^]^

**Scheme 1:**
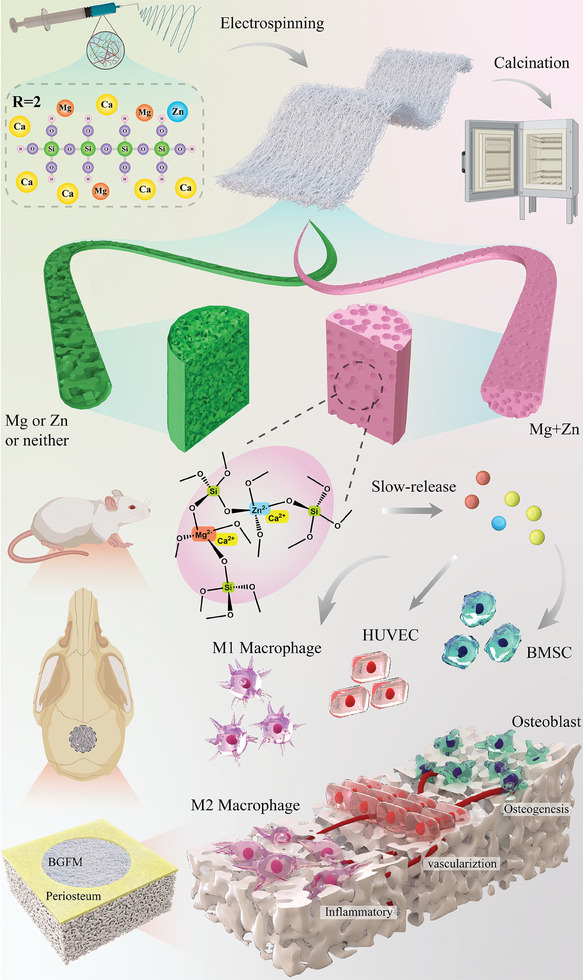
Schematic illustration of the construction of multifunctional bionic periosteum and its ion sustained‐release for bone regeneration.

On the other hand, given that most electrospun bioceramic fiber membranes from sol‐gel solutions rely on polymer binders, eliminating the organic component is crucial during thermal processing.^[^ ^20^ ^]^ Therefore, the fibers become uneven and defective, exhibiting deteriorative mechanical properties.^[^ ^21^ ^]^ Minimizing the amount of organic material incorporated can enhance the mechanical characteristics of bioceramic fibers. According to Sakka et al.’ researches, in an alkoxysilane solutions reaction system, when the molar ratio of H_2_O/Silicon (R) is ≈2 under appropriate acidic environment, the hydrolysis and condensation of alkoxysilane lead to Si–O–Si linear chains (**Scheme** **1**) and consequently great spinnability without polymer binder.^[^ ^22^ ^]^

Conversely, addition of a small amount of polymer binder does not destroy the strength of the fiber greatly, but introduces a porous structure through the principle of phase separation.^[^ ^23^ ^]^ During the electrospinning (ES) process, the phase separation process may occur between the polymer and the inorganic precursor at a critical state, forming the polymer‐inorganic phase separation system. Multi‐porous inorganic fiber materials are obtained after post‐treatment, which was conducive to degradation.^[^ ^24^ ^]^ Using a concentrated inorganic precursor solution with minimal polymer assistance to achieve phase separation can potentially yield a highly robust and flexible bioactive glass fiber membrane (BGFM) with porous architecture.

In this study, a partial hydrolysis method was adopted to prepare BGFM containing Mg and Zn (MgZn‐BGFM), and the phase separation was used to prepare porous structure of the BG fiber. The porous structure can reduce the filling quality and improve the matrix (Si‐O network) degradation. Introduction of magnesium ion (Mg^2+^) and zinc ion (Zn^2+^) altered the property of BG sol, thus affecting the phase separation in the continuous stretching of ES jet. Interestingly, co‐existence of Mg^2+^ and Zn^2+^ can transform the interconnected pores of the BG fiber into closed pores. This shift from interconnected to closed pores reduces the interface area with bodily fluids, effectively limiting the rapid release of calcium ion (Ca^2+^). As shown in Scheme 1, Mg and Zn can form tetrahedral MgO_4_^2^‐and ZnO_4_^2−^ species, respectively, which attract cations (Ca^2+^) to balance the charge. It increases the quantity of BO and locks Ca^2+^ in the BG network, which further inhibits the rapid release of Ca^2+^. By regulating both aspects of structure and component, the MgZn‐BGFM with slower cation dissolution was prepared. The deigned BP with slow release of metal ions regulated the bone immune microenvironment and continuously promoted osteogenic differentiation and vascularization.

## 2 Result and Discussion

### 2.1 Morphology and Pore Structure Analysis

**Figure 1:**
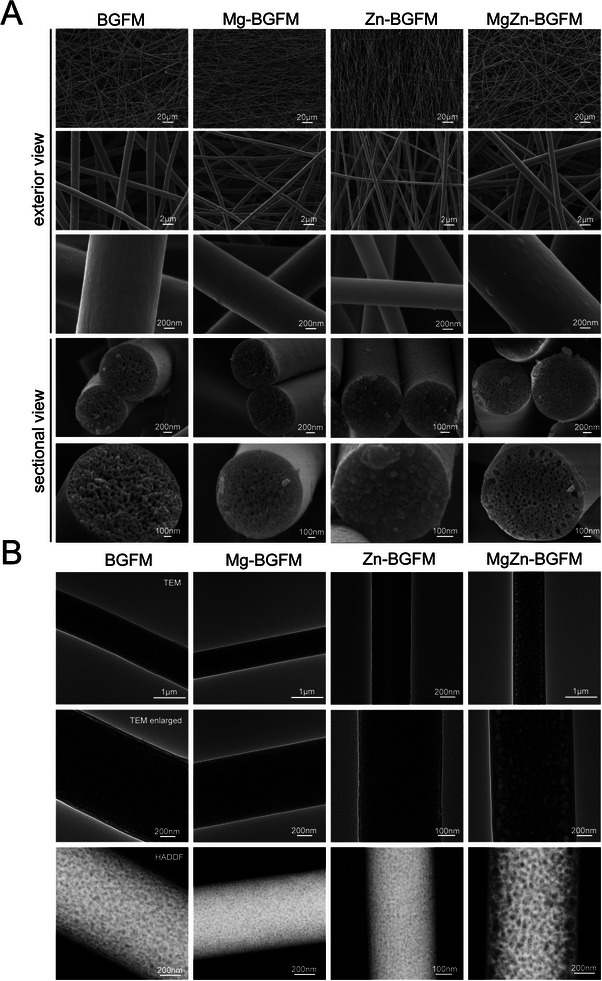
Morphology and pore structure of BPs. A) SEM images of BPs. B) TEM images of BPs.

The BGFM containing Mg was denoted as Mg‐BGFM. The BGFM containing Zn was denoted as Zn‐BGFM. The morphological features of BPs were examined using SEM (**Figure** **1**A). Notably, the fibers of four BPs exhibited good continuity and uniformity, implying that they could form high‐strength and flexible ceramic fiber membranes. The fiber diameter varied with the composition (Figure S1, Supporting Information). With introduction of Mg and Zn, the diameter decreased from 1331.45 nm (BGFM) to 714.56 nm (Mg‐BGFM) and 548.68 nm (Zn‐BGFM), respectively, while the coexistence of Mg and Zn increased the diameter to 1351 nm (MgZn‐BGFM). Analysis of the EDS mapping revealed good uniformity in terms of composition element of BPs (Figure S2, Supporting Information). A sectional view was analyzed to explore the pore structure inside fiber. The pores derived from phase separation during the ES were well‐distributed inside the whole fiber in all BPs. Interestingly, the other three groups exhibited connected pores, while MgZn‐BGFM had closed pores. Moreover, the fibers of Zn‐BGFM contained particles and the gaps between particles formed the pores. The pores in the MgZn‐BGFM material exhibited a larger size (≈105 nm) around the fiber's exterior and a smaller size (≈63 nm) in other regions, as shown in Figure 1B. The TEM results further confirmed the homogeneity of pores in all BPs fibers. Based on the TEM and HADDF images, the porosity was ordered as follows: MgZn‐BGFM > BGFM > Mg‐BGFM ≈ Zn‐BGFM.

**Figure 2:**
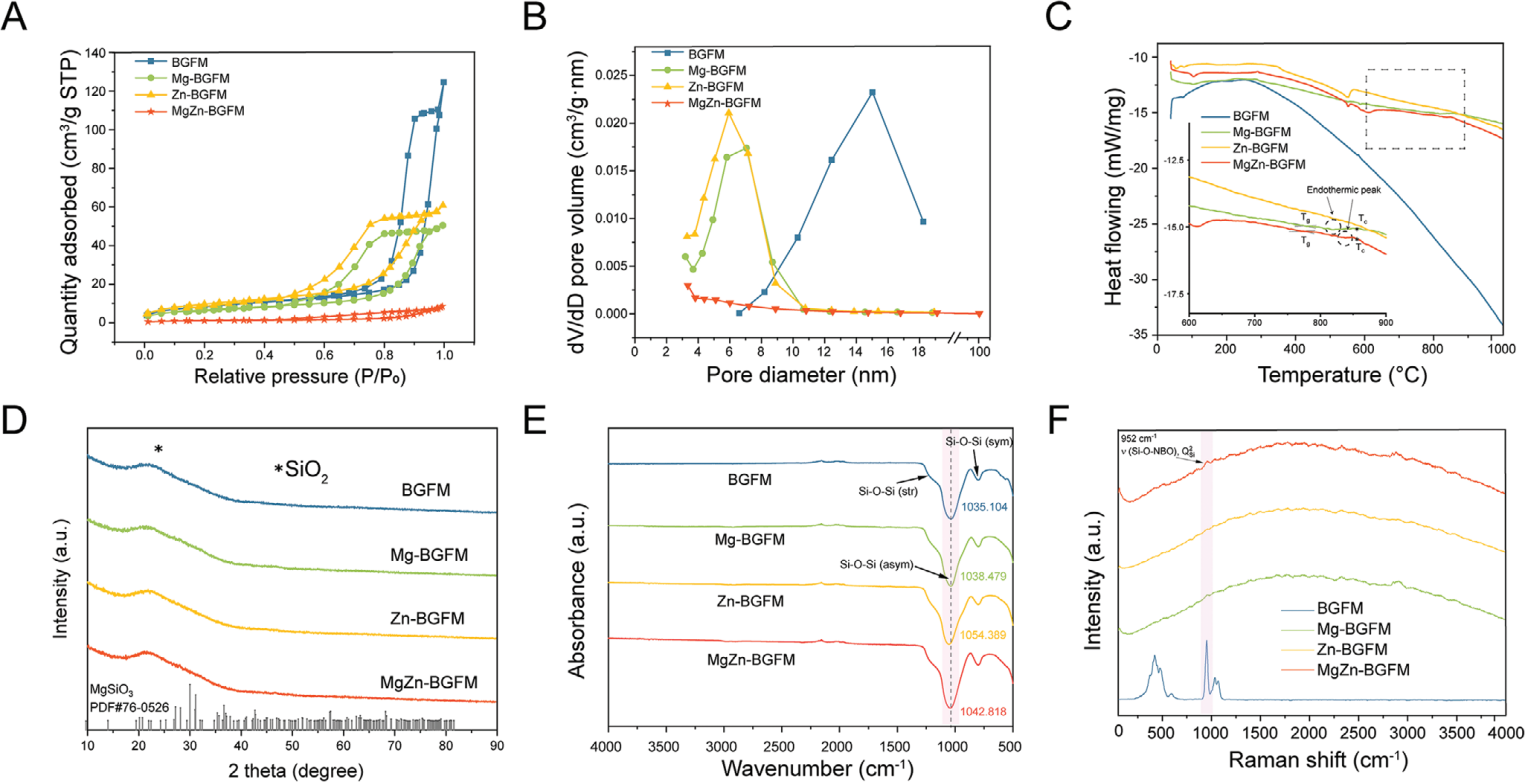
Characterization of BPs. A) N_2_ adsorption/desorption isotherms of BPs. B) Pore size distribution of BPs. C) DSC curves of BPs. D) XRD of BPs calcined at 700 °C. E) FT‐IR spectra of BPs. F) Raman spectra of BPs.

The specific surface area data corresponded with the aforementioned findings (**Figure** **2**A). The MgZn‐BGFM had the lowest specific surface area (3.99 m^2^ g^−1^) compared with others because the closed pores could not be detected by the N_2_ adsorption‐desorption method. The highest specific surface area (35.12 m^2^ g^−1^) of Zn‐BGFM was attributed to the granular structure of fibers. Regarding the similar fiber structure, the BGFM (30.26 m^2^ g^−1^) was higher than Mg‐BGFM (23.62 m^2^ g^−1^) because of the high porosity. This phenomenon was true for the pores size (Figure 2B). Micropores were observed in different regions of the various biomaterials. Specifically, MgZn‐BGFM exhibited micropores of ≈3 nm on the outer surface of the fibers. In contrast, Zn‐BGFM displayed micropores of ≈6 nm within the particles inside the fibers. BGFM revealed micropores of ≈13 nm, while Mg‐BGFM showed micropores of ≈7.5 nm within the interior structure of the fibers.

### 2.2 Phase and Molecular Structure Analysis

Figure 2C indicated that the DSC curves appeared at the glass transition temperature (Tg) of 770 °C for Mg and MgZn‐BGFM. The Tg was followed by an endothermic peak at 820 and 835 °C for Mg and MgZn‐BGFM respectively, and the crystallization temperature (Tc) appeared at 855 °C. This implied the phase change around this temperature.^[^ ^25^ ^]^ Interestingly, the BG particles prepared using sol‐gel method didn't exhibit any Tg or Tc for all 4 compositions (Figure S3, Supporting Information). It indicated the microstructure could also affect the thermodynamic property of BG. The XRD of Mg and MgZn‐BGFM at 800 °C confirmed this result (Figure S4A, Supporting Information). After calcination at 800 °C, the crystalline phase MgSiO3 occurred both in Mg and MgZn‐BGFM. The BPs calcined at 700 °C retained their amorphous phase (Figure 2D). The formation of a crystalline phase could severely compromise the strength and flexibility of the BPs, leading to their fragmentation into smaller pieces (Figure S4B, Supporting Information), thereby limiting their suitability for BP applications. After the introduction of Mg and Zn, the Si‐O‐Si asymmetric stretching vibration in FT‐IR spectra exhibited blue‐shifting to different degrees: Zn‐BGFM > MgZn‐BGFM > Mg‐BGFM > BGFM (Figure 2E). The electron donor group red‐shifted the absorption peak of the chemical bond. Hence, the formation of more NBOs (Ca^2+^ as electron donor group) resulted in enhanced Si‐O‐Si red‐shift. This indicated that Zn inhibited NBO and Mg promoted it to some extent. Nevertheless, the combined effect of Mg and Zn was weaker relative to that of Zn alone. Similarly, the Raman spectra displayed the same trend (Figure 2F). The band at 952 cm^−1^ was assigned to ν (Si‐O‐NBO) belonging to Q2 Si units.^[^ ^19^, ^26^ ^]^ The peak intensities of BPs were ordered similarly as follows: BGFM > MgZn‐BGFM > Mg‐BGFM > Zn‐BGFM. These results demonstrated that the content of NBO in Zn‐BGFM was the least, and was highest in BGFM. In contrast, the combination of Mg and Zn generated a “cocktail” effect, increasing the NBO content compared to Zn.

### 2.3 Ions Release, Mechanical Properties and Bioactivity

**Figure 3:**
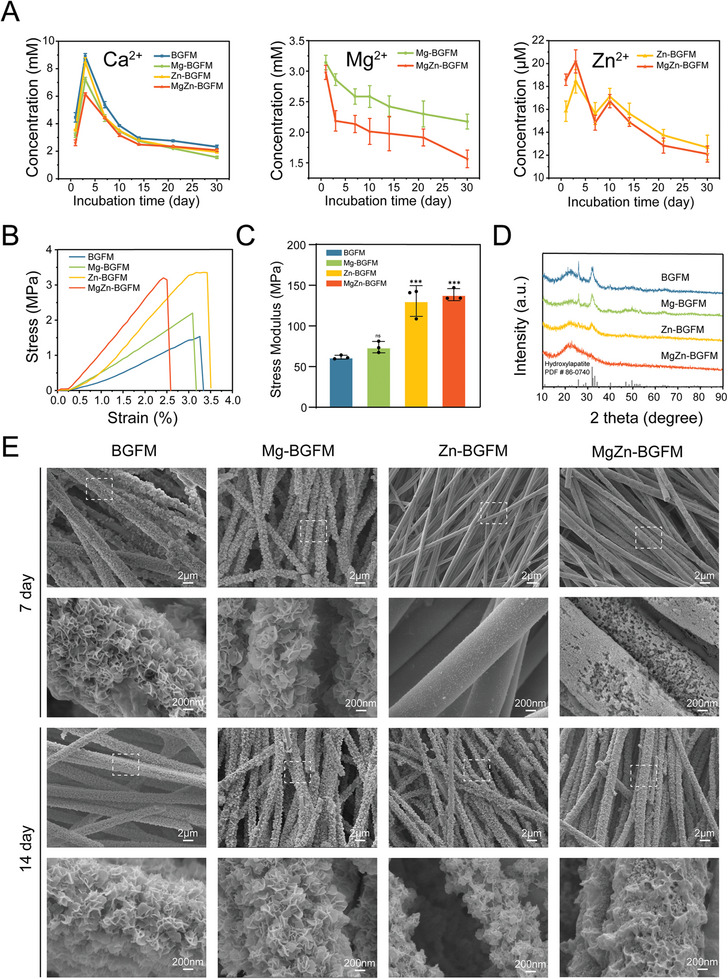
Mechanical properties and bioactivity of BPs. A) Ions release profiles of BPs. B) Tensile stress‐strain curves of BPs. C) Tensile stress modulus of BPs. D) XRD of BPs after immersion in simulated body fluid (SBF) for 14 days. E) SEM images of BPs after immersion in SBF for 7 and 14 days. (n  =  3, *p < 0.05, **p < 0.01, ***p < 0.001).

Assessment of metal ion release was conducted for 30 days (**Figure** **3**A). According to elements composition (**Table** **1**), Ca^2+^ content was in the following order: 20% (BGFM) > 19% (Zn‐BGFM) > 15% (Mg‐BGFM) > 14% (MgZn‐BGFM). At 30 days, the Ca^2+^ concentration of MgZn‐BGFM was higher than that of Mg‐BGFM, which was similar to that of BGFM and Zn‐BGFM. Moreover, the Mg^2+^ concentration of MgZn was consistently lower compared with that of Mg‐BGFM. In addition, the pH change of PBS confirmed the above results (Figure S5, Supporting Information). Among the 4 groups, MgZn‐BGFM exhibited the smallest change, alleviating the inherent drawback of high pH of BG. The above findings show that the release of Ca^2+^ and Mg^2+^ was significantly delayed, and the pH increase was reduced in MgZn‐BGFM. This could be attributed to the closed‐pore structure of the fiber in MgZn‐BGFM and the formation of more negative charged tetrahedral units resulting from the introduction of Mg and Zn. However, there was not significant difference in the Zn^2+^ release of Zn and MgZn‐BGFM. Notably, Zn appeared to play a more important role in glass network formation than Mg. This result agreed with the molecular structure analysis findings (FT‐IR and Raman). The Zn alone was more likely to form negative charged tetrahedral units that locked Ca^2+^ and Mg^2+^ to generate more BO.

**Table 1:** The elements composition data of prepared BPs.

Composition (at %)	Si	Ca	Mg	Zn	P
BGFM	75	20	–	–	5
Mg‐BGFM	75	15	5	–	5
Zn‐BGFM	75	19	–	1	5
MgZn‐BGFM	75	14	5	1	5

The stress‐strain curves and stress modulus are presented in Figure 3B and C, respectively. The Zn (3.36 Mpa) and MgZn (3.20 Mpa) had higher tensile stress than BGFM (1.53 Mpa) and Mg‐BGFM (2.20Mpa). Moreover, the modulus exhibited a similar trend: Zn‐BGFM (130.69 Mpa) and MgZn‐BGFM (138.47 Mpa) > BGFM (61.60 Mpa) and Mg‐BGFM (73.97 Mpa). The Zn‐BGFM fiber exhibited a more robust glass network structure and greater strength, likely due to a higher ratio of BO/NBO. In contrast, the unique closed‐pore topological structure of the MgZn‐BGFM fiber is speculated to account for its properties. The biomineralization was characterized by XRD (Figure 3D) and SEM (Figure 3E). The ability of BGFM to induce the formation of apatite was in the following order: BGFM > Mg‐BGFM >Zn‐BGFM >MgZn‐BGFM. After 7‐day immersion, a large amount of apatite was formed on BGFM and Mg‐BGFM, while nearly no mineral was observed on the two others. Interestingly, the surface of the MgZn‐BGFM fiber peeled off completely and remained intact for Zn‐BGFM. This may imply that MgZn degraded faster than Zn‐BGFM in terms of its chemical components. By the 14th day, some apatite began to form on the Zn and MgZn‐BGFM. As discussed earlier, in the case of Zn‐BGFM, the tendency of Zn to form tetrahedral units appeared to inhibit degradation and apatite induction. Regarding MgZn‐BGFM, the combined effect of its physical structure (closed pores) and chemical composition (metal‐oxygen tetrahedral units) synergistically restrained apatite formation.

### 2.4 Biocompatibility of BPs

To verify the biocompatibility of BPs, the survival status of Mouse mononuclear macrophage (Raw264.7), Bone marrow mesenchymal stem cells (BMMSCs) and Human umbilical vein endothelial cells (HUVECs) on BPs were examined.

**Figure 4:**
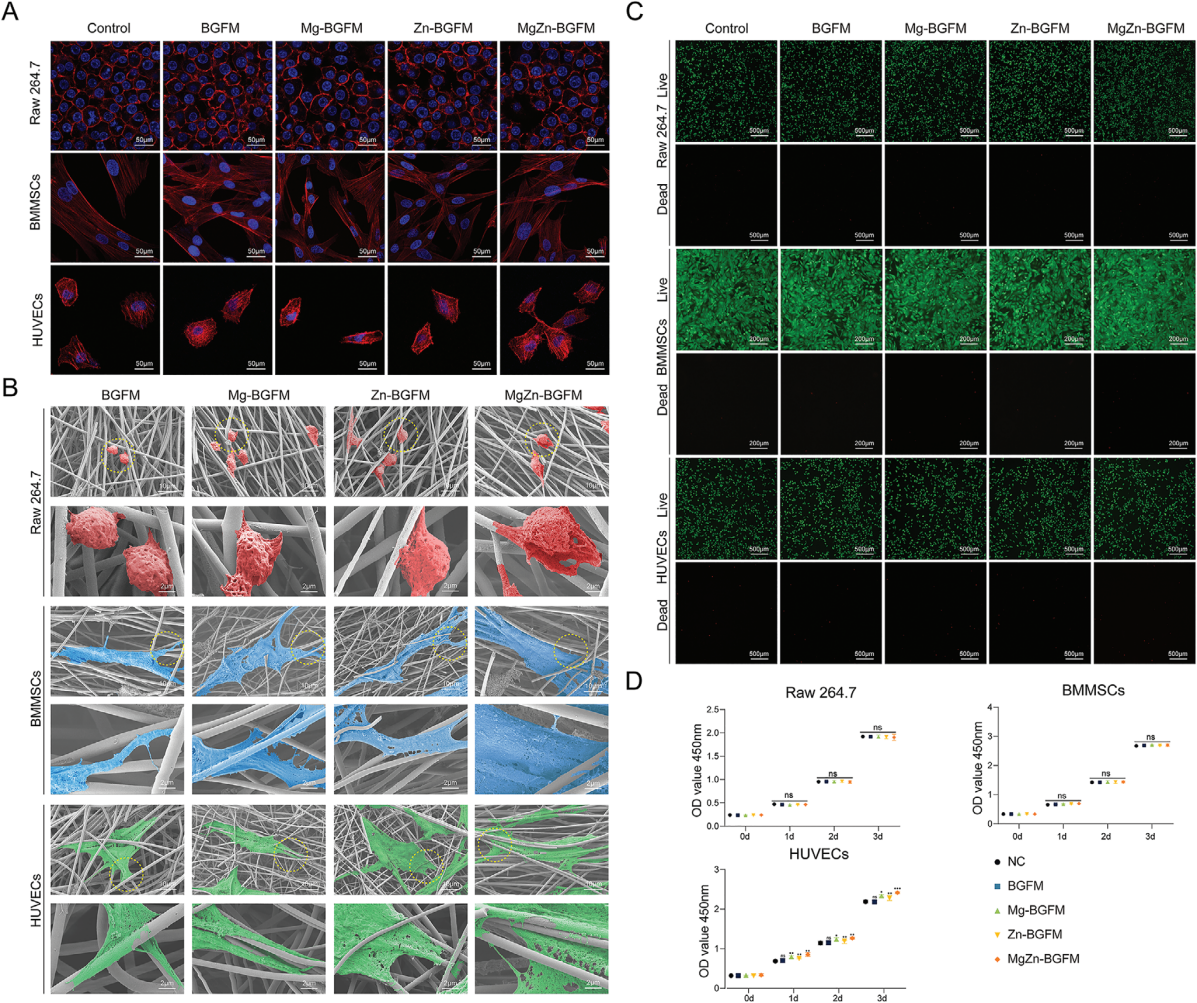
Biocompatibility of BPs. A) The cytoskeleton staining of Raw264.7, BMMSCs and HUVECs on BPs indicating the adhesion state of the cells. B) SEM of the adhesion of Raw264.7, BMMSCs and HUVECs on BPs. C) The living state images of Raw264.7, BMMSCs and HUVECs on BPs. Green fluorescence indicates living cells and red, fluorescent nuclei indicates dead cells. D) OD values of the supernatant of Raw264.7, BMMSCs and HUVECs culture medium demonstrating cell proliferation capacity. (n  =  3, *p < 0.05, **p < 0.01, ***p < 0.001).

Raw264.7, BMMSCs, and HUVECs were individually seeded onto the surface of BP. After 24 h, cytoskeleton staining was conducted using phalloidin. The cytoskeleton plays an important role in cell adhesion and migration.^[^ ^27^ ^]^ Cytoskeletal staining can reflect the biocompatibility of BPs. The findings revealed similar cell morphology between the control group and the BPs group, suggesting no significant difference in cell adhesion and migration status on BPs (**Figure** **4**A). In subsequent tests, we examined growth of cells on the surface of BPs using SEM. The findings were consistent with those of the phalloidin staining, and the three types of cells formed good adhesion with different components (Figure 4B). Chemically active substances can provide favorable conditions for cell adhesion behavior. Mg^2+^ for example, can enhance cell attachment by facilitating binding interactions between the integrin family of cell surface receptors (transmembrane proteins that mediate cell adhesion) and their ligand proteins.^[^ ^28^ ^]^ Studies have indicated that a Mg^2+^ concentration of 10 mM can promote the adhesion and differentiation of osteoblasts.^[^ ^29^ ^]^ Zn^2+^ can also promote cell extension of pseudopodia, which promotes the initial adhesion and diffusion of cells.^[^ ^30^ ^]^

After 72 h of culturing, Raw264.7, BMMSCs, and HUVECs were subjected to the live/dead staining. The results revealed that the majority of cells were viable, with only a small minority exhibiting signs of cell death. No significant differences were observed between the groups (Figure 4C). Similar results were obtained from apoptosis tests (Figure S6, Supporting Information). The results suggested that the Ca, Zn and Mg ions released by the material were not toxic to cells.

The biocompatibility of a material is often assessed both in terms of its cytotoxicity and cell proliferation. In this study, the results of CCK‐8 assay showed that OD values of Raw264.7 and BMMSCs groups were comparable after 3 days of continuous culture. Notably, the activity of HUVECs increased following the inclusion of Zn and Mg, with Mg demonstrating a comparatively stronger promoting effect (Figure 4D). This underscores the favorable living conditions provided by BPs for cells.

### 2.5 Macrophage Polarization of BPs Regulation In Vitro

Bone comprises various intricate tissues, and their interplay is pivotal in preserving the equilibrium of bone turnover. The involvement of macrophages in bone turnover is paramount. Macrophages undergo polarization in response to diverse stimuli. The M1 type is pro‐inflammatory and unfavorable for osteogenic differentiation, whereas the M2 type, characterized as anti‐inflammatory, has been demonstrated to modulate the differentiation of bone marrow mesenchymal stem cells, thus exerting a significant influence on bone nonunion and osteoporosis.^[^ ^31^ ^]^ Therefore, we explored whether we could provide a favorable environment for bone regeneration by regulating the polarization of macrophages to promote osteogenic differentiation.

**Figure 5:**
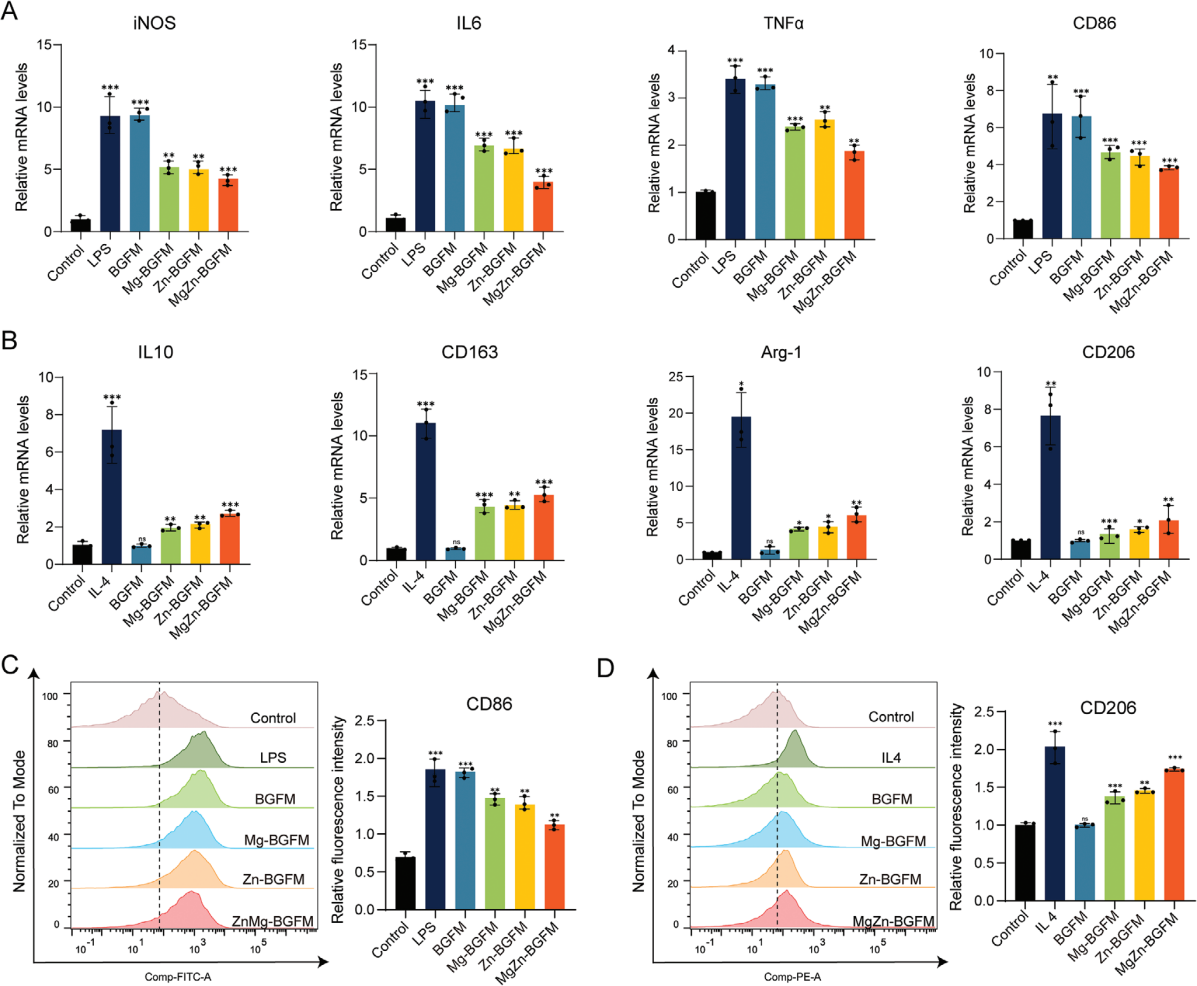
Macrophage polarization of BPs regulation in vitro. A) The relative expression levels of mRNA related to M1 macrophages, including iNOS, IL6, TNF‐α and CD86. B) The relative expression levels of mRNA related to M2 macrophages including IL10, CD163, Arg‐1 and CD206. C) The mean fluorescence intensities of CD86 (M1 marker) measured by flow cytometry. D) The mean fluorescence intensities of CD206 (M2 marker) measured by flow cytometry. (n  =  3, *p < 0.05, **p < 0.01, ***p < 0.001).

To verify the effect of BPs on the polarization of macrophages, by RT‐PCR and flow cytometry tests were conducted to evaluate the polarization of macrophages. iNOS, TNF‐α, IL6 and CD86 are the hallmark indicators of M1 type. To verify the inhibitory effect on M1 type polarization, Raw264.7 was induced to M1 type polarization by LPS incubation for 24 h, and then cultured with the extract. It was observed that LPS stimulation, the expression levels of iNOS, TNF‐α, IL6 and CD86 increased significantly, suggesting successful induction of M1 type. However, this change was not observed after addition of the BGFM extract, but the expression decreased after addition of MgZn‐BGFM (**Figure** **5**A). IL‐10, CD163, Arg‐1, and CD206 are the characteristic indicators of M2 phenotype. To verify the M2 macrophage, we induced Raw264.7 with interleukin 4 (IL‐4) stimulation and then subjected them to other treatments. The results demonstrated a significantly higher expression level of IL‐10, CD163, Arg‐1, and CD206 in the IL‐4 group than in the control group, indicating successful induction of M2‐type macrophages. While the IL‐4 group displayed the highest expressions of IL‐10, CD163, Arg‐1, and CD206, the other four groups (excluding the BGFM group) showed lower but significantly elevated levels compared to the control group (Figure 5B). Flow cytometry corroborated these findings, indicating that the MgZn‐BGFM group had the lowest average fluorescence intensity of CD86 and the highest for CD206 (Figure 5C and D).

Further analysis of the experimental results indicated that Zn and Mg modulated macrophage polarization, and Zn had a stronger effect than Mg. Our experiments showed that Mg^2+^ and Zn^2+^ released by BP promoted macrophage maturation. Previous studies have also confirmed that proteins related to macrophage maturation, such as protein kinase C (PKC) and collagenase MMP‐9, bind to Zn^2+^ to execute their functions.^[^ ^32^ ^]^ Low dose of Zn^2+^ was found to be more effective in regulating macrophages, and the concentration of Zn^2+^ in the range of 11.25–45 µM promoted M2‐type polarization while inhibiting M1‐type polarization.^[^ ^33^ ^]^ Zn and MgZn‐BGFM could release Zn^2+^ continuously in vitro, and the concentration could be maintained at 10–20 µM for 30 days. Similarly, Mg^2+^ demonstrated dose‐dependent immunomodulation within the range of 0.4–20 mM. Mg and MgZn‐BGFM could release Mg^2+^ continuously in vitro, and the concentration could be maintained at 1.5–3.5 mM for 30 days. Research has demonstrated that Mg^2+^ can facilitate the polarization of macrophages into M2 type via the TRPM7 channel, thus facilitating the recruitment and osteogenic differentiation of BMMSCs.^[^ ^34^ ^]^ Our results showed that the Zn and Mg released by MgZn‐BGFM promoted the polarization of macrophages toward M2 type, which provided a favorable immune environment for osteogenic differentiation.

### 2.6 Osteogenesis Promotion of BPs In Vitro

**Figure 6:**
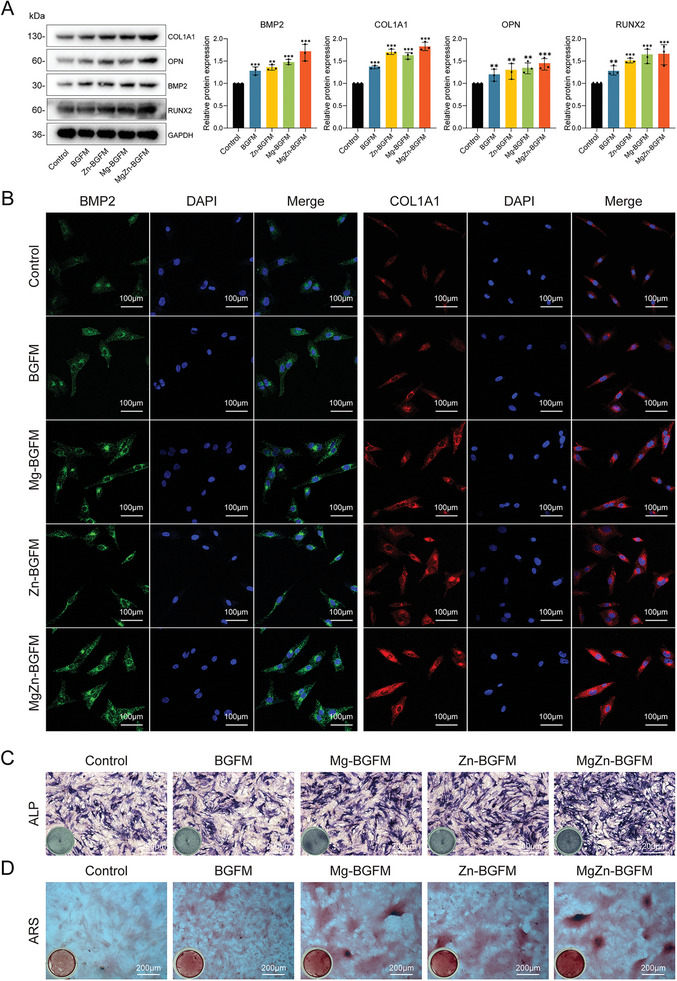
Osteogenesis promotion of BPs in vitro. A) During osteogenic differentiation, the protein expression level and statistical data of osteoblast‐related genes in each group. B) Representative images of immunofluorescence staining in each group during osteogenic differentiation. Green represents BMP2 and red represents COL1A1, with blue indicating the nucleus. C) During osteogenic differentiation, representative images of ALP staining in each group, with dark colors representing positive cells. D) ARS representative images of each group during osteogenic differentiation. (n  =  3, *p < 0.05, **p < 0.01, ***p < 0.001).

To investigate the effect of BPs on osteogenic differentiation of bone marrow mesenchymal stem cells, we evaluated the expression of osteogenic differentiation‐related markers such as bone morphogenetic protein‐2 (BMP2), collagen type I (COL1A1), Runt‐associated transcription factor (Runx2) and osteopontin (OPN) in vitro. The results showed that the expression levels of the above proteins were increased after the addition of Ca, Zn and Mg ions, and this effect was most significant in the MgZn‐BGFM group (**Figure** **6**A). COL1A1 and BMP2 participates in the regulation of collagen formation and osteoblast maturation, and hence the formation of new bone. Thus, we employed immunofluorescence tests to detect the expression levels of COL1A1 and BMP2, and obtained similar results (Figure 6B). Alkaline phosphatase (ALP), a widespread membrane‐bound glycoprotein produced by active osteoblasts, is an important marker of osteoblast differentiation and early bone mineral formation on biomaterials in vitro. Mineralized nodules are not only a sign of osteoblast differentiation and maturation, but also the main morphological feature of osteoblast function.^[^ ^35^ ^]^ To assess osteoblast differentiation, common techniques include observing mineralized nodules. Consequently, ALP staining and alizarin red S staining (ARS) were conducted, yielding consistent results. The MgZn‐BGFM group exhibited the highest proportion of ALP‐positive cells, while the number of calcium nodules was highest in alizarin red S staining (Figure 6C and D).

Previous researches indicated that Ca^2+^, Mg^2+^ and Zn^2+^ of MgZn‐BGFM promoted osteogenic differentiation of BMMSCs in vitro.^[^ ^36^ ^]^ The three ions can not only promote osteogenic differentiation independently, but also promote each other and play a synergistic role. Ca^2+^ not only enhances the expression of OPN, OCN, and BMP‐2, but also regulates the expression of transcription factors associated with osteogenic differentiation, such as Runx‐2 and osterix.^[^ ^37^ ^]^ Both Mg and Zn facilitated osteogenic differentiation, with the effect of Mg^2+^ being stronger. Osteogenesis consumes significant energy, and the body's metabolic state directly affects osteogenic activity.^[^ ^38^ ^]^ Mg was found to significantly improve the level of cell energy metabolism, thereby promoting osteogenesis via the AKt‐glycolytic‐MRs2‐mitochondrial axis. Mg can play a synergistic role with calcium, such as enhancing the osteogenic induction capacity of BMP2, activating calcium ion channels on cell membranes and promoting calcium deposition.^[^ ^39^ ^]^ Zn modulates healthy bone growth and development. The Zn receptor (ZnR)/Gpr39 is required for normal bone matrix deposition in osteoblasts.^[^ ^40^ ^]^ Studies have shown that Zn enhances osteogenic differentiation of BMMSCs by activating PKA signaling pathway and insulin‐like growth factor‐1 (IGF‐1) activity.^[^ ^41^ ^]^ Moreover, Mg and Zn can potentiate the effects of vitamin D in promoting osteogenic differentiation.^[^ ^42^ ^]^ In our study, whether it is the expression of osteogenic genes, or ARS and ALP staining, BP can promote osteogenic differentiation.

### 2.7 Angiogenesis Promotion of BPs In Vitro

In vitro tests showed that MgZn‐BGFM group combined the advantages of both Mg‐BGFM group and Zn‐BGFM group without significant side effects. Therefore, in vivo experiments, we focused on the effect of MgZn‐BGFM group on bone regeneration.

**Figure 7:**
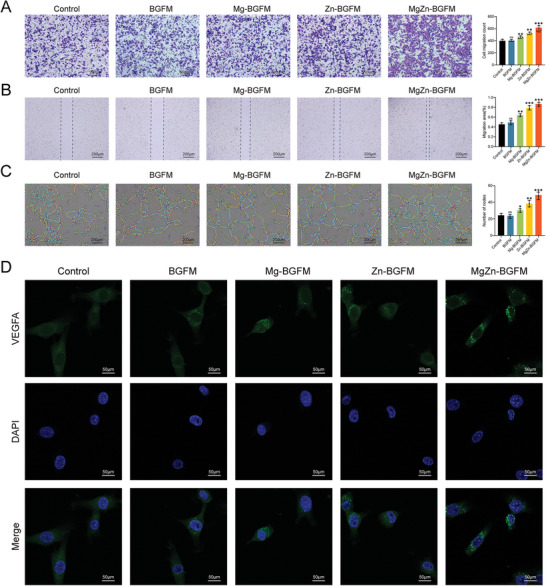
Angiogenesis promotion of BPs in vitro A) The transwell test showing the ability of vascular endothelial cells to migrate. Purple represents completed migration cells. B) Scratched wound assay and quantitative analysis of vascular endothelial cells. C) The representative images of the angiogenesis test. The yellow line represents the closed loop formed and the red dot represents the formed nodes, which demonstrated the angiogenesis ability. D) Representative images of vascular endothelial cells in each group by immunofluorescence staining. Green represents VEGFA and blue represents nucleus. (n  =  3, *p < 0.05, **p < 0.01, ***p < 0.001).

The blood supply in the periphery of bone defects influence the formation of new bone because bone regeneration concurrently occurred with blood vessel formation.^[^ ^43^ ^]^ To test our hypothesis, we examined the effect of BPs on angiogenesis in vitro. The migration of endothelial cells is the initial stage of angiogenesis. Thus, we performed scratch tests and cross‐hole tests to examine the migration ability of endothelial cells in all groups. The results showed that the area of cell migration and the number of transpores increased after the addition of Zn and Mg, implying that Zn and Mg promote the migration of endothelial cells to different degrees, and this effect was most significant in the MgZn‐BGFM group (**Figure** **7**A and B). Moreover, Zn had a stronger effect than Mg in promoting endothelial cells migration. Prior research has indicated that Zn can augment the expression of matrix metalloproteinases, thus amplifying the degradation of the vascular basement membrane, which is crucial for endothelial cell migration.^[^ ^44^ ^]^ Subsequently, the effect of BPs on angiogenesis was simulated in vitro and the results showed that the largest number of nodes and rings was formed in the MgZn‐BGFM group (Figure 7C). As an active regulator of bone development, VEGFA can indirectly link angiogenesis to osteogenesis owing its effects on endothelial cells, and can also directly regulate osteoblasts.^[^ ^45^ ^]^

Studies have shown that the Zn‐Mg alloy can upregulate the expression of angiogenesis‐related genes via the FGF/FGFR pathway.^[^ ^46^ ^]^ Mg has also been reported to induce endothelial cells leading to the production of nitric oxide and activation of VEGFA eventually inducing angiogenesis.^[^ ^47^ ^]^ Therefore, we conducted immunofluorescence staining to examine the expression of VEGFA, and the results showed that the addition of Zn and Mg increased VEGFA expression (Figure 7D). The experiments indicated that BPs facilitate the formation of new blood vessels, which not only supply necessary nutrients for bone regeneration but also directly enhance osteogenic differentiation.

### 2.8 Bone Regeneration Promotion of BPs In Vivo

**Figure 8:**
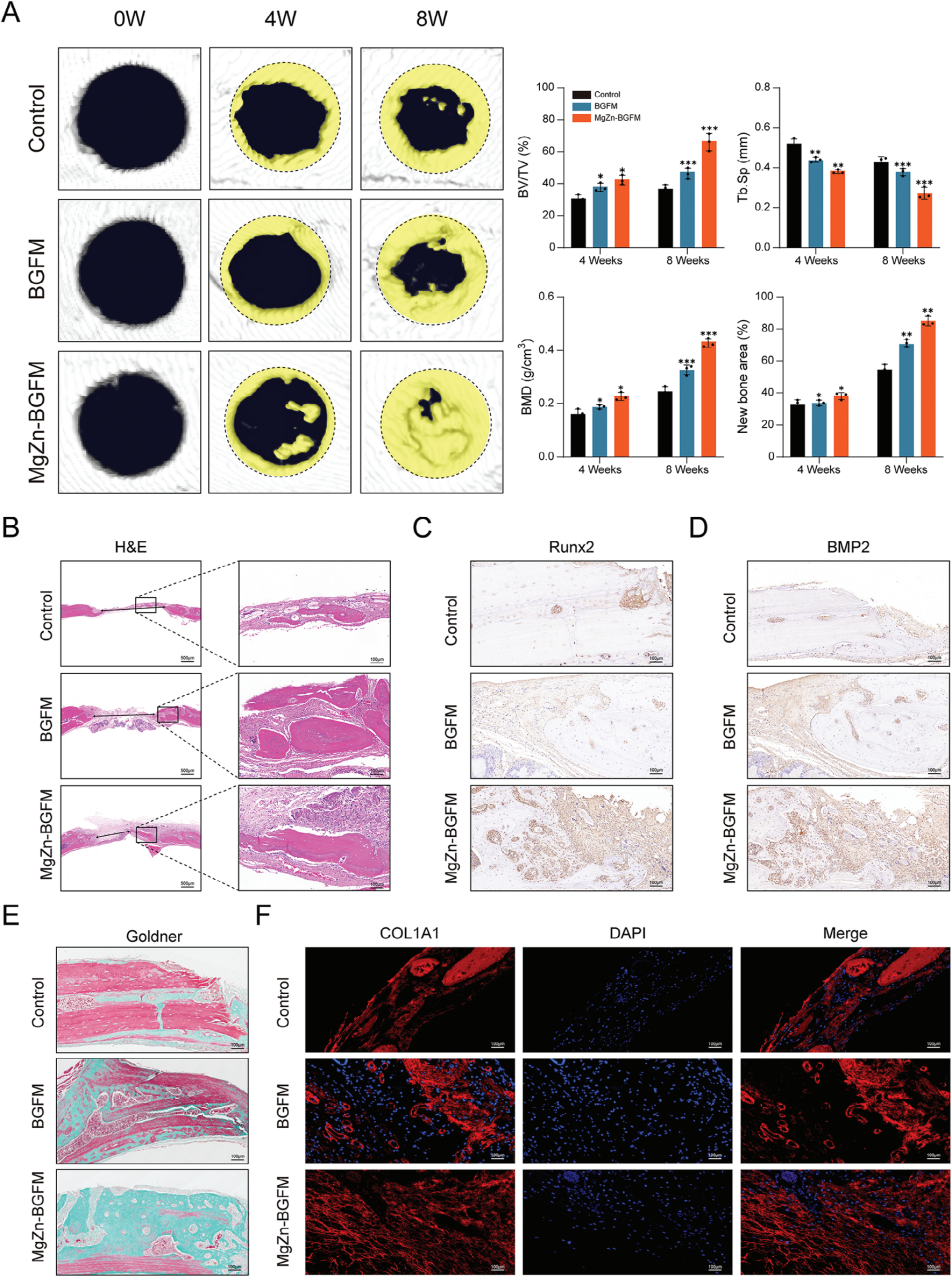
Bone regeneration promotion of BPs in vivo. A) Representative Micro‐CT images of skull defects of the same rat in each group at 4 weeks and 8 weeks. The statistical data shows the parameters of bone volume fraction (BV/TV), trabecular separation (Tb.sp), bone mineral density (BMD), and new bone area. B) Representative H&E staining images of skulls of different rat groups, with black bidirectional arrows indicating unhealed bone defects. C) and D) Representative images of Runx2 and BMP2 Immunohistochemistry (IHC) in different groups of skull defect tissue. E) Representative images of Goldner three‐color staining in different groups of skull defect tissue. The green part represents new bone. F) Representative images of COL1A1 immunofluorescence staining in different groups of skull defect tissue. Red represents type I collagen and blue represents the nucleus. (n  =  3, *p < 0.05, **p < 0.01, ***p < 0.001).

To evaluate the role of BPs in bone repair in vivo, an animal model of bone defect was established. Micro‐CT showed that the MgZn‐BGFM group exhibited the best healing at 4 and 8 weeks after surgery (**Figure** **8**A). The mean bone mineral density was ≈0.433 g cm^3^, and the mean bone healing area ratio was ≈85.20%, which exceeded that of the control group and BGFM group.

To further investigate the effect of BPs on skull defect repair, histological evaluation was performed using the H&E staining and Goldner tricolor staining tests. Analysis of the H&E staining images showed more new bone plates and bone islands in the MgZn‐BGFM group (Figure 8B). Similarly, the Goldner tricolor staining test showed more collagen deposition in the MgZn‐BGFM group (Figure 8E). In addition, COL1A1 immunofluorescence staining on bone tissue yielded similar results (Figure 8F).

Based on the effects of Runx2 and BMP2 on osteogenesis, immunohistochemical staining was further performed on bone tissues, which showed that the proportion of positive cells in MgZn‐BGFM group was significantly higher than that in the control group (Figure 8C and D).

These results suggest that BPs facilitated bone regeneration and collagen deposition in vivo.

### 2.9 M2 Macrophages and Vascular Regeneration Promotion of BP In Vivo

**Figure 9:**
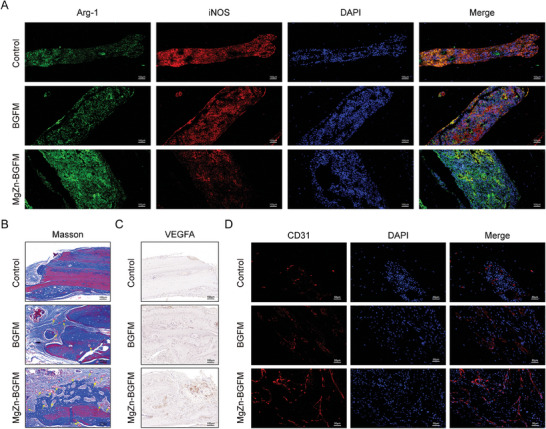
M2 macrophages and vascular regeneration promotion of BPs in vivo. A) Representative images of macrophage‐related immunofluorescence staining of rat skull tissue. Green represents Arg‐1 and red represents iNOS, with blue indicating the nucleus. B) Representative Masson staining images of rat cranial tissue. Blue represents new collagen and yellow arrow indicates new blood vessels at the defect. C) Representative images of VEGFA IHC staining of rat skull tissue. D) Representative images of CD31 immunofluorescence staining of rat skull tissue. Red represents CD31 and blue represents the nucleus. (n  =  3, *p < 0.05, **p < 0.01, ***p < 0.001).

In further experiments, we explored the polarization of macrophages and neovascularization in the bone defect. Macrophage infiltration was examined through tissue immunofluorescence. It was observed that the expression of Arg‐1 in skull tissue was higher in the MgZn‐BGFM group, while the expression of iNOS was decreased in the MgZn‐BGFM group compared with the control group (**Figure** **9**A). This result was the same as in vitro experiment, indicating that M2 macrophage infiltration near bone defect increased after implantation of BPs.

Results of the Masson staining shown in Figure 9B demonstrated that new blood vessels were formed at the bone defect (yellow arrow), providing favorable conditions for bone regeneration. As mentioned above, VEGFA in not only an indicator of vascular endothelial cells function, but also acts as a mediator for angiogenic osteogenic coupling. Immunohistochemical analysis showed that the expression of VEGFA was highest in MgZn‐BGFM group (Figure 9C). Subsequently, tissue immunofluorescence staining was conducted to measure the expression of CD31, which is expressed at the tight junctions between endothelial cells. The results revealed that the expression of CD31 was highest in MgZn‐BGFM group (Figure 9D).

Collectively, these findings indicated that BPs could promote bone defect repair in vivo, owing to its ability to regulate immune invasion and vascular regeneration. The favorable immune microenvironment not only enhances osteogenic differentiation, but also promotes neovascularization. The newly formed blood vessels supply nutrients needed to sustain osteogenic differentiation and the proliferation of osteoblasts.

### 2.10 BP Affects Macrophage Polarization by Regulating JAK‐STAT Signaling Pathway

**Figure 10:**
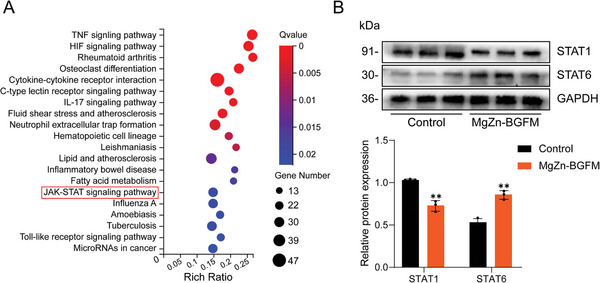
The effect of MgZn‐BGFM on macrophage polarization by regulating JAK‐STAT signaling pathway. A) The top 20 representative up‐regulated or down‐regulated pathways analyzed by KEGG pathway method. B) The protein expression level and statistical map of STAT1 and STAT6 in each group. (n  =  3, *p < 0.05, **p < 0.01, ***p < 0.001).

To further explore the molecular mechanism by which BP regulate the polarization of macrophages, we conducted transcriptomic sequencing of Raw264.7 cultured on BP. Next, investigated that activated pathways through the KEGG pathway analysis. **Figure** **10**A displays the top 20 activated pathways. Based on previous studies, we selected on the JAK‐STAT signaling pathway. STATs are transcription factors that bind to target gene promoters and modulate macrophage polarization. Currently, eight types of STATs have been detected, among which STAT1 is known to promote the secretion of pro‐inflammatory cytokines by macrophages and induce macrophage polarization toward M1 type, while STAT6 promotes the polarization of M2 type macrophages.^[^ ^48^ ^]^

Subsequently, Western blotting technology was conducted to quantify the expression of STAT1 and STAT6. It was observed that BP promoted STAT6 expression and inhibited STAT1 expression (Figure 10B). This may form the molecular mechanism underlying the effects of BP in promoting polarization of M2‐type macrophages.

## 3 Conclusion

In this study, a novel BG fiber BP was designed. Introduction of Mg^2+^ and Zn^2+^ modified the phase separation during the ES jet stretching process, resulting in structural transition from connected to closed pores in the BG fiber. This change reduced the rapid release of metal ions, decreased the filling quality and improved degradation. In addition, Mg and Zn formed negative charged metal‐oxygen tetrahedral units (MgO_4_^2−^ and ZnO_4_^2−^) in the Si‐O network, thereby firmly locking in the positive charged metal ions such as Ca^2+^ and inhibiting their release. Finally, through structural and component designs, the porous MgZn‐BGFM with delayed metal ion release was prepared and applied for the bionic replacement of periosteum.

In vitro experiments have demonstrated that synthetic periosteum fosters the polarization of M2‐type macrophages by releasing Mg^2+^ and Zn^2+^, creating a conducive immune environment for bone regeneration. This process is associated with the JAK‐STAT signaling pathway. In addition, BP can directly enhance osteogenic differentiation and angiogenesis, facilitating bone regeneration. In vivo, we found that BP stimulated bone regeneration. In summary, we designed a BP with slow release of metal ions for regulating bone immune microenvironment and enhancing osteogenic differentiation and vascularization, thereby accelerating bone regeneration.

## 4 Experimental Section

### Materials

Tetraethyl orthosilicate (TEOS, 98.0%), calcium nitrate tetrahydrate (Ca(NO_3_)_2_·4H_2_O, ≥ 99.0%), magnesium nitrate hexahydrate (Mg(NO_3_)_2_·6H_2_O, ≥ 99.0%), nitric acid (HNO_3_, 65.0%–68.0%) and absolute ethanol (ETOH, ≥ 99.7%) were purchased from Sinopharm Chemical Reagent Co., Ltd. Zinc nitrate hexahydrate (Zn(NO_3_)_2_·6H_2_O, 99.99%), triethyl phosphate (TEP, 99.5%) and polyvinyl butyral (PVB, M.W. 170 000–250 000) were purchased from Shanghai Aladdin Biochemical Technology Co., Ltd. A calcium (Ca) colorimetric assay kit, Mg colorimetric assay kit and Zn colorimetric assay kit were purchased from Elabscience Biotechnology Co., Ltd. (Wuhan, China). Mouse monocyte macrophage leukemia cells (RAW264.7) and human umbilical vein vascular endothelial cells (HUVECs) were purchased from Shanghai Zhong Qiao Xin Zhou Biotechnology Co., Ltd. Phosphate‐buffered saline (1×), dexamethasone (≥98.0%), β‐glycerophosphate sodium salt hydrate (≥ 98.0%), DAPI, TRITC‐phalloidin, RIPA cell lysate, Alizarin Red S, Masson Trichrome Stain Kit and Goldner Trichrome Stain Kit were purchased from Beijing Solarbio Science & Technology Co., Ltd. DMEM, penicillin‒streptomycin and EDTA‐free trypsin were purchased from Gibco (Shanghai, China). Fetal bovine serum (FBS) was purchased from Shanghai Wenren Biotechnology. The ascorbic acid (99.0%), calcein/PI cell viability/cytotoxicity assay kit, cell counting kit‐8 (CCK‐8), BCA protein assay kit, BCIP/NBT alkaline phosphatase color development kit and lipopolysaccharide (LPS) were purchased from Beyotime Biotechnology. IL‐4 was purchased from Nearshore Protein (China). Annexin V‐FITC/PI Apoptosis Detection Kit, a FastPure Cell/Tissue Total RNA Isolation Kit V2, and a HiScript III 1st Strand cDNA Synthesis Kit were purchased from Vazyme Biotech Co., Ltd. APC‐conjugated anti‐mouse F4/80, PE‐conjugated anti‐mouse CD206, and FITC‐conjugated anti‐mouse CD86 were purchased from Biolegend (USA). Matrix gels were purchased from Corning (USA). The first antibody used in the western blot assay was purchased from Cell Signaling Technology. All chemicals were used as received without further purification.

### Fabrication of BPs

BGFM containing Mg and Zn was fabricated via the sol‐gel method with silicon source partial hydrolysis. First, 15 ml of TEOS and 0.77 ml of triethyl phosphate was dissolved in 7 ml of EtOH, after which 3 g of Ca(NO_3_)_2_·4H_2_O, 1.157 g of Mg(NO_3_)_2_·6H_2_O and 0.268 g of Zn(NO_3_)_2_·6H_2_O were added. After stirring for half an hour, 0.95 ml of H_2_O was added to the solution to satisfy R ≈ 2. Subsequently, 100 µL HNO_3_ (1 N) was added to the above solution, and stirring continued for another 2 h. Afterwards, 2 ml PVB‐ETOH solution (6% w/v) was added to the above sols to introduce phase separation for electrospinning. The obtained sols were stirred for 36 h in an ice bath, and persistent volatilization occurred to increase the viscosity of the sols. When the total volume decreased to 20 ml, the aged hybrid solutions were loaded into a plastic syringe equipped with a metallic needle (21 gauge) to spin the fiber. A high‐tension field (16 kV) was applied to the metal needle and plate collector. The obtained precursor of the bioactive glass membrane (PBGFM) was transferred into an oven at 50 °C for 12 h to volatilize the residual solvent. Finally, the samples were heated to 700 °C at a heating rate of 1 °C min^−1^ and annealed for 5 h in air.

The different BGFMs were prepared according to the above methods, and their compositions are shown in Table 1. BG particle precursors with the same composition were prepared using the sol‐gel method, and the equivalent PVB was added. Without the ES process, the sol slowly transformed into a gel after 2 days, and the BG particle precursor was obtained after drying at 50 °C.

### Characterization

The morphologies and elemental compositions of the BGFMs were investigated using field scanning electron microscopy (FE‐SEM, JSM‐7800, JEOL, Japan) with energy dispersive X‐ray spectroscopy (EDS). The pore size and distribution were further observed by transmission electron microscopy (TEM, JEM F200, JEOL, Japan). The phase compositions were measured by X‐ray diffraction (XRD, D/MAX‐Ultima IV, Rigaku, Japan). The specific surface area and pore structure of the BGFMs were determined via N2 adsorption‐desorption tests (ASAP 2460, Micromeritics, USA).

The functional groups and chemical structures of the BGFMs were examined using FT‐IR (Nicolet iS50, Thermo Scientific, American). The weight loss and possible phase transition during the calcination of the PBGFMs were measured using thermogravimetry and differential scanning calorimetry (TG‐DSC, TGA/DSC 3+, LER TOLEDO, Switzerland) at a heating rate of 10 °C min^−1^. The chemical structure of the BGFMs was tested by Raman spectroscopy (LabRAM HR evolution, Jobin Yvon‐Horiba, France) over the 50–4000 cm^−1^ range.

### Ions Release

The metal ion release of the BGFMs was measured by colorimetry as follows: 50 mg of BGFMs was soaked in 10 ml of DMEM, 10 ml of medium was removed at specific time points (1, 3, 7, 10, 14, 21, and 30 days) for assay analysis to determine the concentration of Ca, Mg and Zn in the medium, and 10 ml of fresh DMEM was added to the immersed samples. The BGFMs were added to PBS at 5 mg/ml. The pH of the solution was measured within 80 h using a pH meter (CT‐6020, KEDIDA, China).

### Mechanical Test

The mechanical properties of the BGFMs were analyzed using an electronic fiber strength tester (LLY‐06). The strength of BPs with a thickness of 50 ± 10 µm and a size of 10 mm* 4 mm was measured.^[^ ^49^ ^]^

### Bioactivity Test

SBF was prepared according to the usual procedure^[^ ^50^ ^]^ using chemicals purchased from Sinopharm Chem. Reagents Co., Ltd. The bioactivity of BPs was tested in vitro by immersing sample fragments in SBF at 37 °C to monitor the formation of HCA for different time intervals (7 and 14 days). The test parameters were determined by the equation Vs = Sa/10, where Vs is the volume of the SBF (ml) and Sa was the apparent surface area of the specimen (mm^2^). The biomineralization behavior was analyzed using SEM and XRD.

### Cell Culture

RAW264.7 and HUVECs were purchased from Zhongqiao Xinzhou Company, and high‐glucose DMEM supplemented with 10% fetal bovine serum (FBS, UE500, SERANA, Shanghai Wenren Biotechnology Co., Ltd) and 1% penicillin/streptomycin solution was used according to the manufacturer's instructions. BMMSCs were isolated from the femurs and tibias of 8‐week‐old male rats after anesthesia. Low‐glucose DMEM was used, and osteogenesis was induced by the addition of 10 mM sodium β‐glycerophosphate, 10 nM dexamethasone, and 50 µg mL^−1^ ascorbic acid.

### Cytoskeleton Staining

RAW264.7 cells, BMMSCs and HUVECs were cultured on different bioglass fiber membranes. The cells were fixed using 4% paraformaldehyde. Cytoskeletal staining was performed using phalloidin. A confocal laser scanning microscope (Zeiss Germany) was used for visualization.

### Live/Dead Staining

After 72 h of treatment, the cells were inoculated in 24‐well plates and incubated with calcein AM and propidium iodide according to the manufacturer's instructions to stain live and dead cells. The sections were then placed under an inverted fluorescence microscope (Zeiss, Germany) for observation.

### Apoptosis Detection

The cells were digested using EDTA‐free trypsin, washed 3 times with PBS, and treated with an Annexin V‐EGFP/PI Apoptosis Detection Kit (Cat No.40302; Yeasen, Shanghai, China) according to the manufacturer's instructions. The proteins were detected by flow cytometry (Beckman, USA), and the results were analyzed using FlowJo_V10.

### Cell Proliferation Assay

The cells were inoculated in 96‐well plates and cultured with different BPs extracts. Then, 10% CCK‐8 reagent was added on days 0, 1, 2 and 3 of culture, and after culturing for 30 min, the absorbance of the supernatant at 405 nm was analyzed using a microplate reader (PerkinElmer, Finland).

### RNA Extraction and RT‒PCR

RNA was extracted from cells using a FastPure Cell/Tissue Total RNA Isolation Kit V2. cDNA was synthesized from extracted mRNA using a HiScript III 1st Strand cDNA Synthesis Kit according to a standard procedure. Hifair III One Step RT‐qPCR SYBR Green Kit (Cat No.11143, Yeasen, Shanghai, China) was used for quantitative real‐time reverse transcription polymerase chain reaction (RT‒PCR). Qualitative analysis was performed using a photocycler 480 system (Roche Applied Science, Germany). The specific sequences of primers used in this study are shown in Table S1 (Supporting Information).

### Western Blotting Analysis

Cellular proteins were extracted using RIPA cell lysis buffer. The protein concentration was determined using a BCA kit. Protein samples were mixed with 5× SDS‐PAGE upsampling buffer and boiled in a water bath for 10 min. Proteins were separated using a rapid PAGE gel preparation kit and transferred to PVDF membranes. The primary antibodies used for this study were anti‐COL1A1, anti‐BMP2, anti‐Runx2, anti‐OPN, anti‐STAT1, anti‐STAT6 and anti‐GAPDH, and all the antibodies were diluted according to the manufacturer's instructions.

### Immunofluorescence

Cells were inoculated in 24‐well plates, and after the cells had adhered to the wall, the cells were rinsed in PBS and fixed with 4% paraformaldehyde for 15 min, permeabilized with 0.5% trilatone X‐100 for 10 min and then blocked with 20% goat serum for 30 min. The cells were incubated for 12 h at 4 °C with specific antibodies according to the manufacturer's instructions and for 30 min with fluorescent secondary antibodies protected from light. Finally, the results were visualized using a confocal laser scanning microscope (Zeiss, Germany) after restaining with DAPI.

### Alkaline Phosphatase Staining

After 7 days of induction of BMMSCs using osteogenic medium, alkaline phosphatase staining was performed using the BCIP/NBT Alkaline Phosphatase Color Development Kit. The staining solution was prepared by adding BCIP, NBT and working solution according to the recommended procedure. The mixture was then incubated at room temperature in the dark for 60 min. The reaction was terminated by removing the staining solution and washing with water 1–2 times.

### Alizarin Red Staining

Twenty‐one days after the induction of BMMSCs using osteogenic medium, calcium salt deposition in each group was assessed using an alizarin red staining kit according to the manufacturer's instructions.

### Macrophage Induction and Characterization

RAW264.7 cells were induced using 200 ng mL^−1^ lipopolysaccharide for 12 h to induce polarization toward the M1 phenotype. RAW264.7 cells were induced with 10 ng mL^−1^ interleukin 4 for 12 h to induce polarization toward the M2 phenotype. To assess macrophage polarization, the cells were digested with EDTA‐free trypsin. The cells were washed with PBS, incubated for 30 min with the primary antibody and detected using a flow cytometric analyzer (Beckman USA), and the results were analyzed using FlowJo_V10. The antibodies used were APC‐conjugated anti‐mouse F4/80, PE‐conjugated anti‐mouse CD206, and FITC‐conjugated anti‐mouse CD86.

### Cell Migration Assay

The cells were inoculated in 6‐well plates, cultured until they were fully confluent, and then prestarved for 24 h before the experiment. Cells were scratched with a 200 µL pipette to make equal‐width wounds, washed with PBS, added to low‐serum medium, and left to migrate for 24 h. Cell migration was photographed using a microscope.

Transwell chambers were placed in 24‐well plates with basal medium in the upper layer, and complete medium was added as a chemoattractant in the lower layer. The cells were prestarved for 24 h. Then, the cell suspension was placed in the upper layer of medium, and incubation was continued for 12 h. The cells in the chambers were fixed in 4% paraformaldehyde and then stained with crystal violet. The cells were removed from the upper layer and photographed randomly using a microscope.

### Tube Formation Assay

To simulate blood vessel formation in vitro, a matrix gel was placed in a 96‐well plate, and when it was allowed to solidify, prestarved cells were inoculated in the 96‐well plate at a density of 15 000 per well and placed under a microscope to observe blood vessel formation after 3 h.

### Animal Model

Eight‐week‐old male SD rats were selected for the animal model. Rats were housed in the Experimental Animal Centre of Qilu Hospital (Approval No. Dwll‐2023‐136), Shandong University, in an air‐conditioned room at 23–25 °C with a light‒dark cycle time of 12 h, where they had adequate access to water and food. The rats were randomly divided into 3 groups with 3 in each group: sham operation group, BGFM group and MgZn‐BGFM group. the sham operation group, the BGFM group and the MgZn‐BGFM group. After the rats were anesthetized, the skin was cut, and the skull was exposed. After the periosteum was excised, a 5 mm diameter defect was made on the skull surface. The BPs was used to cover the bone defect, and the incision was sutured. All procedures and instruments used were performed in a sterile environment.

### Micro‐CT Scanning and Analysis

Rats were anesthetized and sacrificed. The skull was dissected and fixed with 4% paraformaldehyde. Tissues were scanned using a high‐resolution micro‐CT scanner (Perkin Elmer, Japan). The parameters were adjusted to a voltage of 90 kV, 88 µA and a resolution of 7 µm per pixel. Skyscan NRecon software (PerkinElmer, Japan) was used to reconstruct the images, and CTVox software (PerkinElmer, Japan) was used to analyze the parameters of the samples.

### Histological Staining

Rat skulls were fixed in 4% paraformaldehyde for 24 h and decalcified using EDTA for 2 weeks. Sections were made according to previous studies. H&E staining was performed according to the recommended procedure. After deparaffinization and hydration, the sections were stained with hematoxylin for 5 min and 1% eosin Y for 10 min. A Masson Trichrome Stain Kit and a Goldner Trichrome Stain Kit were used to stain the collagen according to the manufacturer's instructions. Slides were photographed using a microscope (Zeiss, AXIO).

### Immunohistochemistry and Immunofluorescence Staining

For immunohistochemical staining analysis, dewaxed sections were treated with 3% H_2_O_2_ for 5 min followed by 5% BSA for 10 min. The sections were then incubated with primary antibody at 4 °C overnight. Subsequently, they were incubated with a biotin‐coupled secondary antibody and visualized using the streptavidin‐biotin staining technique. The cell nuclei were stained with hematoxylin, and the slides were photographed using a microscope (Zeiss, AXIO).

For immunofluorescence staining, trypsin was used for antigen repair, and 3% H_2_O_2_ was used to eliminate endogenous peroxidase activity. The tissues were incubated with the primary antibody at 4 °C for 12 h. Fluorescently labeled goat anti‐rabbit IgG secondary antibody was incubated at room temperature for 1 h. Images were captured using fluorescence microscopy (Ti2‐U, Nikon, Japan). The primary antibodies used were anti‐CD31, anti‐COL1A1, anti‐iNOS and anti‐Arg‐1, which were diluted in accordance with the manufacturer's instructions.

### Statistical Analysis

All the data were shown as the mean ± standard deviation (SD). All experiments were repeated at least three times. Student's *t*‐test was used for comparisons between two independent groups, and one‐way analysis of variance (ANOVA) was used for comparisons between multiple groups. Statistical significance was set at *p* < 0.05.

## Conflict of Interest

The authors declare no conflict of interest.

## Supporting information

Supporting Information

## Data Availability

Research data are not shared.
