# Supplementary material for: Multifunctional Bionic Periosteum with Ion Sustained‐Release for Bone Regeneration
Source: Adv Sci (Weinh). 2024 Sep 3;11(39):2403976. doi: 10.1002/advs.202403976 (PMC11497021; doi:10.1002/advs.202403976)
Supplement: Supplementary file 1 — Supporting Information [file ADVS-11-2403976-s001.pdf]

ADVANCED SCIENCE
Open Access

Supporting Information

for *Adv. Sci.*, DOI 10.1002/advs.202403976

Multifunctional Bionic Periosteum with Ion Sustained-Release for Bone Regeneration

Junjie Mao, Zhenqian Sun, Shidong Wang, Jianqiang Bi, Lu Xue, Lu Wang, Hongliang Wang, Guangjun Jiao* and Yunzhen Chen**

Supporting Information

Multifunctional bionic periosteum with ion sustained-release for bone regeneration

Junjie Mao#, Zhenqian Sun#, Shidong Wang#, Jianqiang Bi, Lu Xue, Lu Wang, Hongliang Wang, Guangjun Jiao*, Yunzhen Chen**

Table S1. the primer sequences for each primer used in the qRT-PCR.

Genes	Forward primer	Reverse primer
Arg-1	AGGCGCTGTCATCGATTTCT	TGGAGTCCAGCAGACTCAAT
IL10	AGGCGCTGTCATCGATTTCT	TGGAGTCCAGCAGACTCAAT
iNOS	GCGCTCTAGTGAAGCAAAGC	AGTGAAATCCGATGTGGCCT
TNF-a	CTCAGCGAGGACAGCAAGG	AGGGACAGAACCTGCCTGG
IL6	CAACGATGATGCACTTGCAGA	TGTGACTCCAGCTTATCTCTTGG
GAPDH	CTTCATTGACCTCAACTACATGGTCTA	GATGA CAAGCTTCCC ATTCTCAG
Cd206	CTCTGTTCAGCTATTGGACGC	CGGAATTTCTGGGATTCAGCTTC
CD86	TTGTGTGTGTTCTGGAAACGGAG	AACTTAGAGGCTGTGTTGCTGGG
CD163	GGGTCATTCAGAGGCACACTG	CTGGCTGTCCTGTCAAGGCT

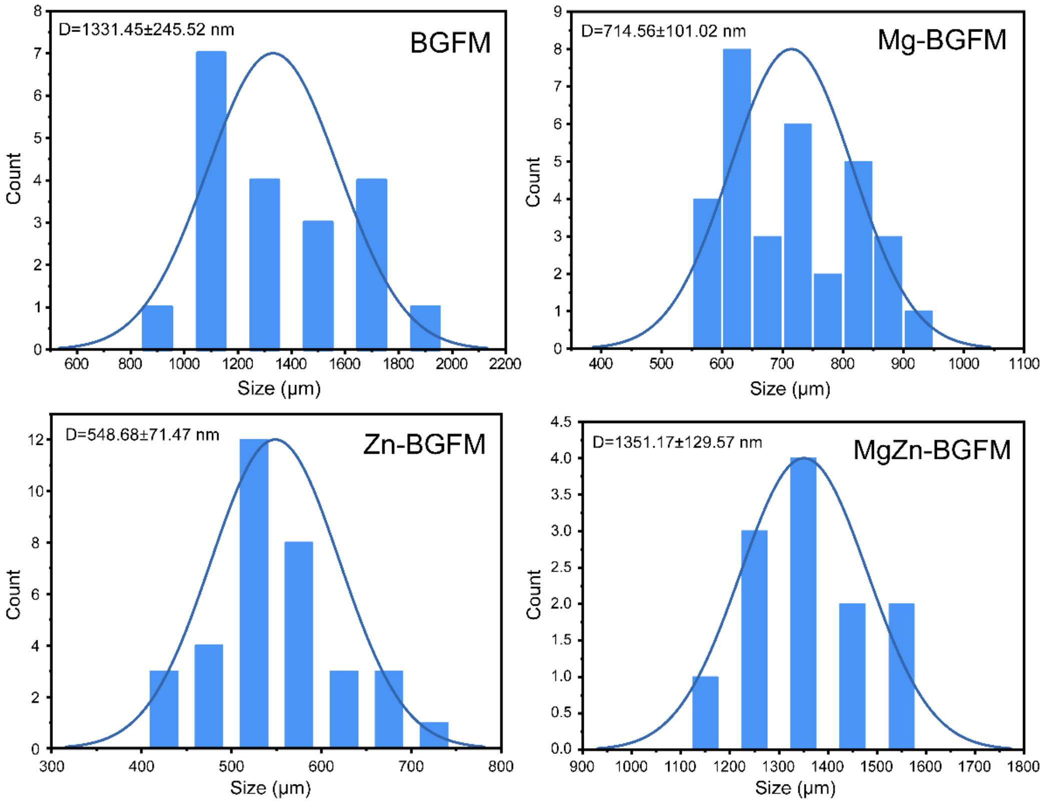

Figure S1. Fiber of BPs diameter distribution.

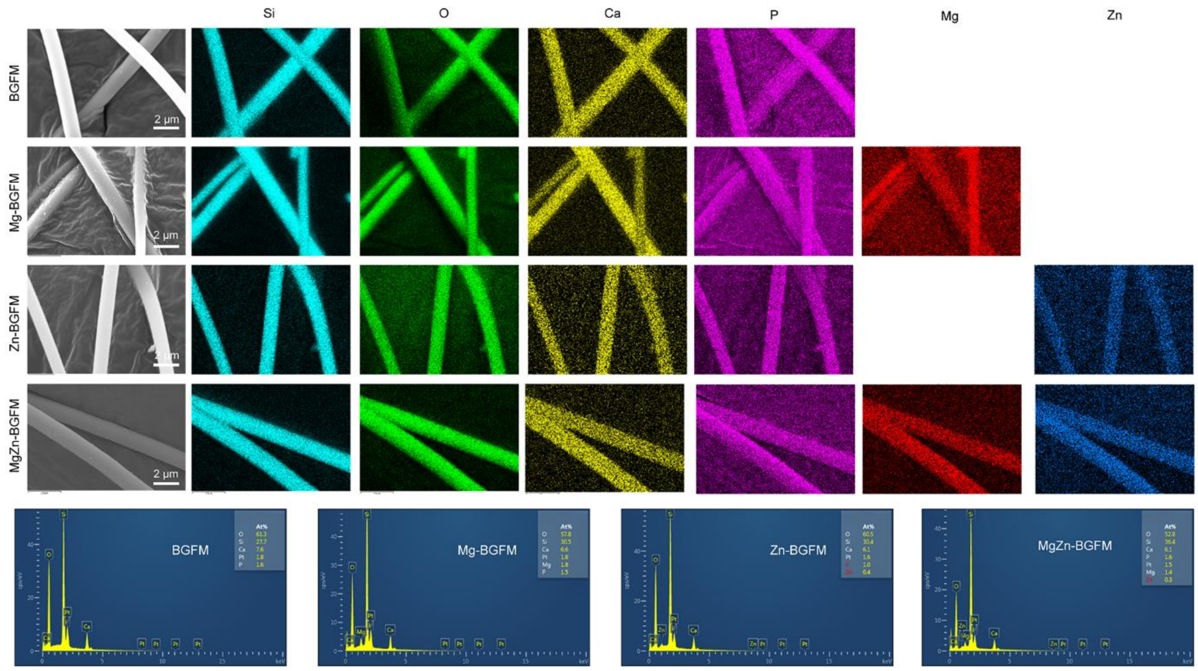

Figure S2. EDS mapping of BPs fibers.

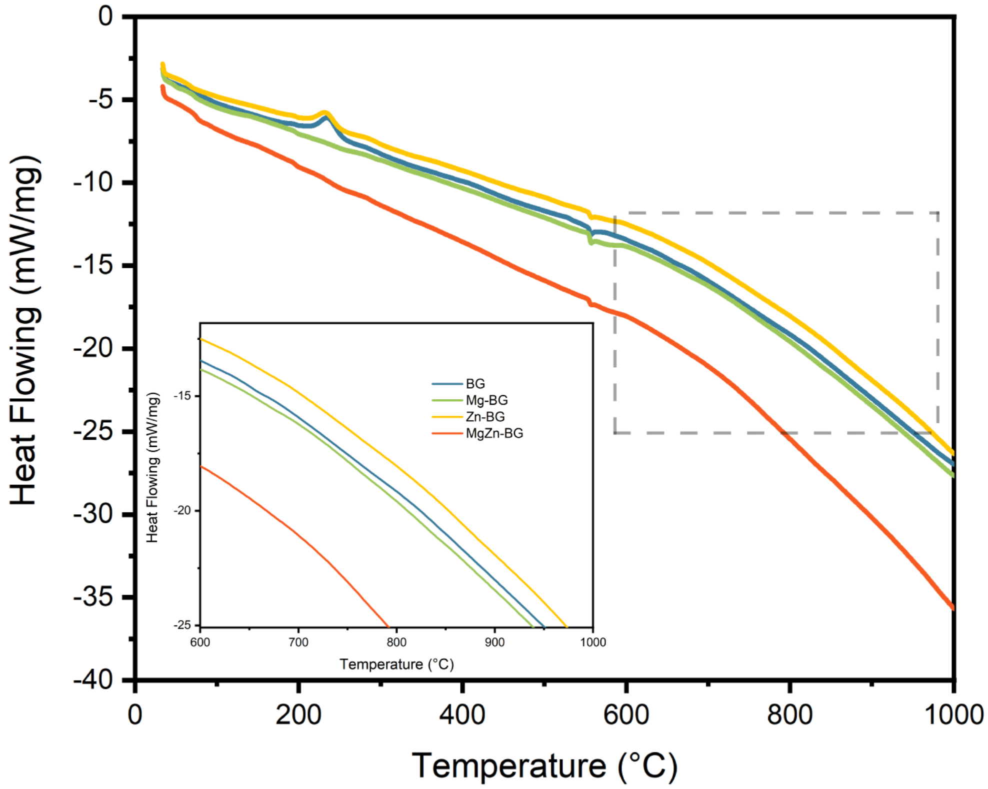

Figure S3. DSC of BG particles precursor.

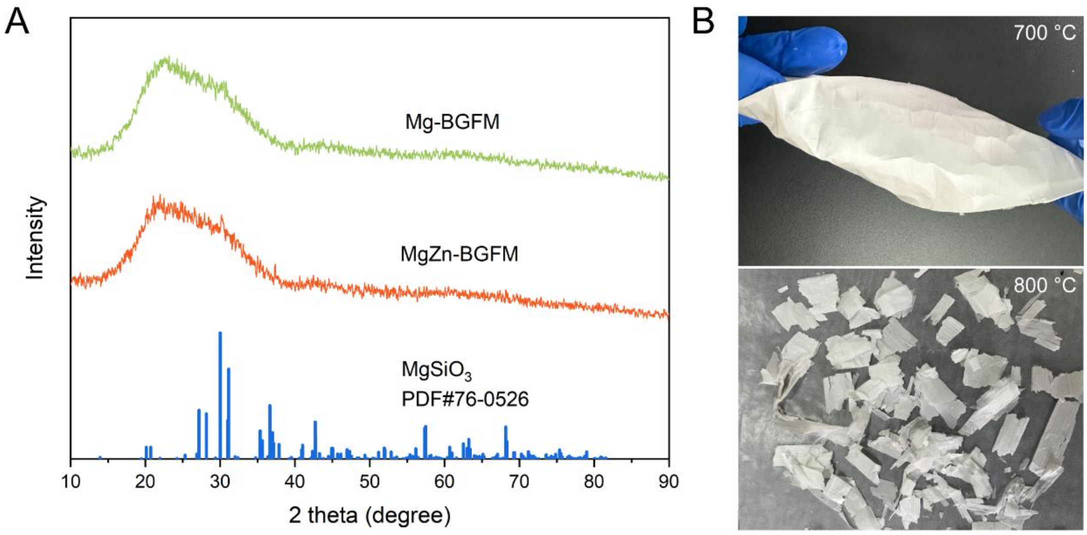

Figure S4. A) XRD of Mg-BGFM and MgZn-BGFM calcined at 800 °C. B) The optical images of MgZn-BGFM calcined at 700 °C and 800 °C.

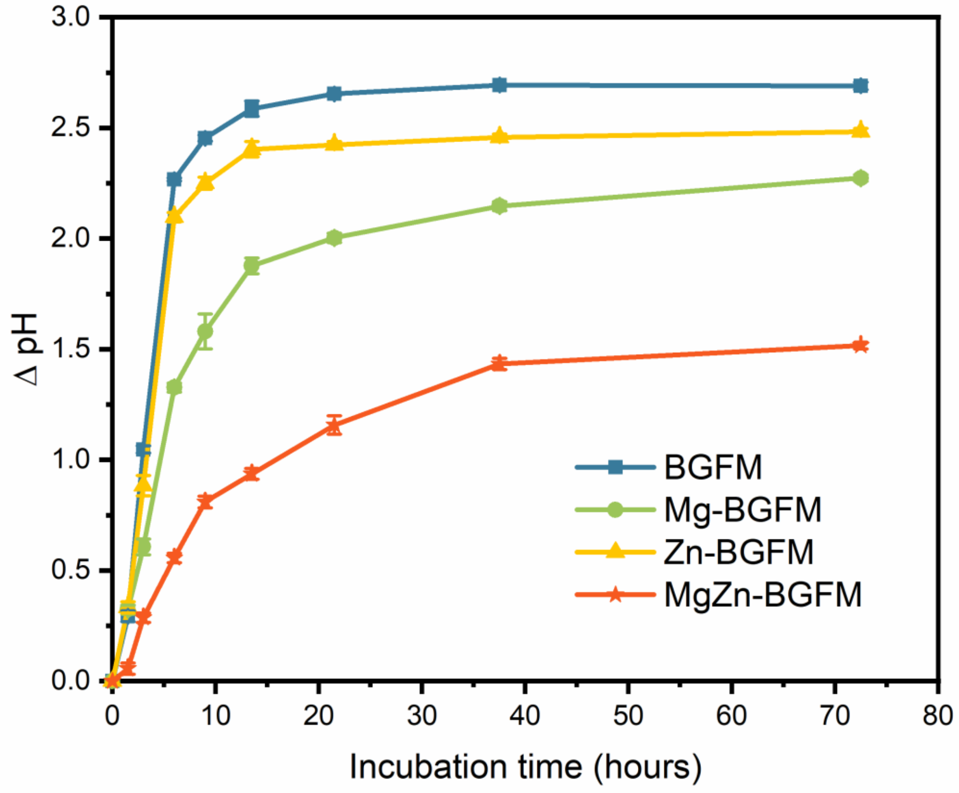

Figure S5. pH value change of BPs soaked in PBS.

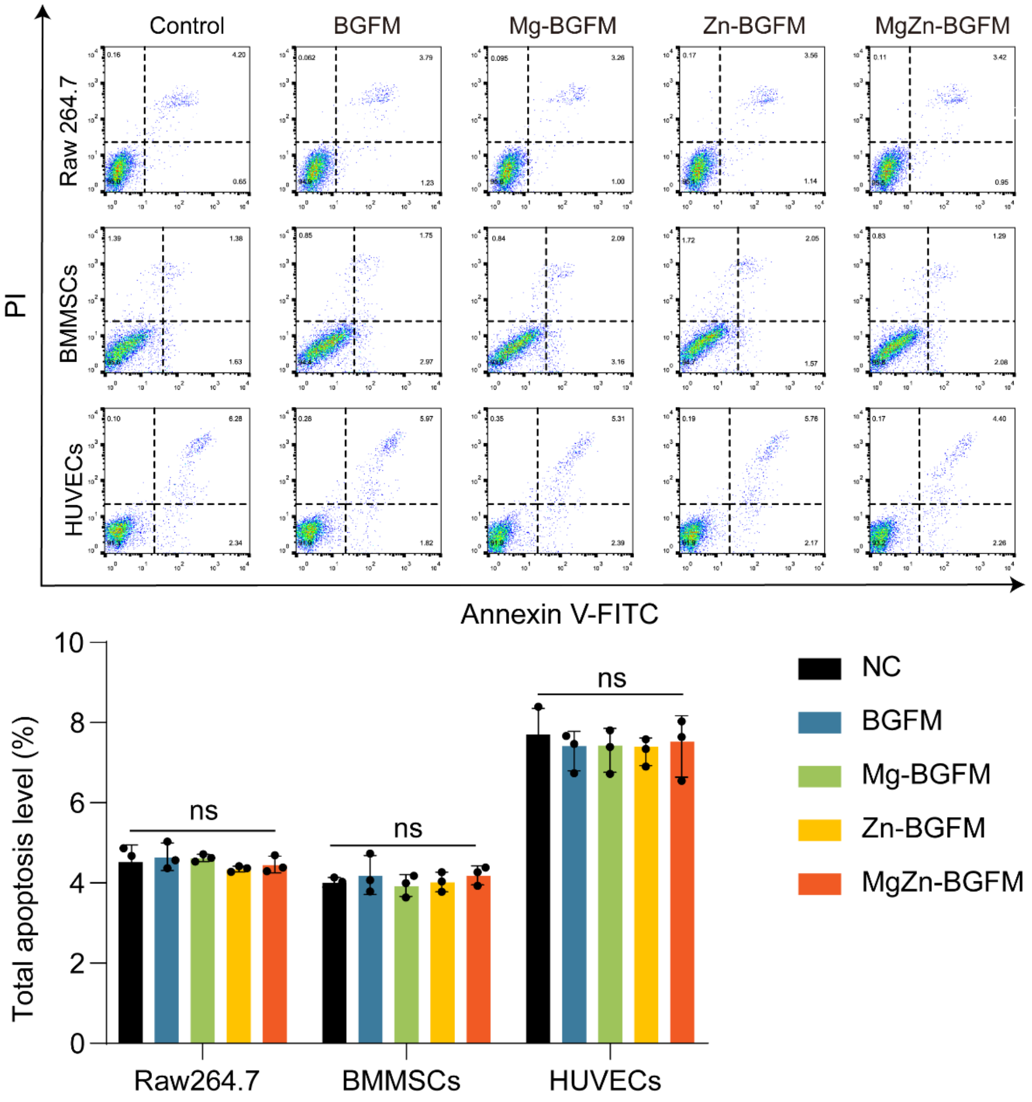

Figure S6. The apoptosis levels of three kinds of cells were measured by flow cytometry.
